# In Situ Synthesis of (Mo,Cr)Si_2_ Composites by Spark Plasma Sintering

**DOI:** 10.3390/ma17164105

**Published:** 2024-08-19

**Authors:** Yue-Yao Wang, Guo-Hua Zhang

**Affiliations:** State Key Laboratory of Advanced Metallurgy, University of Science and Technology Beijing, Beijing 100083, China; wangyueyao9@163.com

**Keywords:** molybdenum disilicide, (Mo,Cr)Si_2_ composites, oxidation behavior, mechanical properties

## Abstract

This research investigated the impact of Cr content on the properties of (Mo,Cr)Si_2_ composites. Composites with CrSi_2_ molar fractions ranging from 0% to 10% were fabricated using spark plasma sintering (SPS). The study undertook a systematic analysis of the surface morphology, phase composition, mechanical properties, and high-temperature oxidation resistance of the sintered samples across different compositions. Notably, the (Mo_95_,Cr_5_)Si_2_ composite sintered at 1400 °C exhibited enhanced properties, including a Vickers hardness of 11.6 GPa, a fracture toughness of 4.6 MPa·m^1/2^, and a flexural strength of 397 MPa. Upon oxidation at 1500 °C, the (Mo,Cr)Si_2_ composites formed a protective oxide layer comprised of SiO_2_ and Cr_2_O_3_. It was found that the generation and thickening of the protective oxide layer was promoted by the addition of moderate amounts of Cr to MoSi_2_.

## 1. Introduction

Owing to its excellent performance, including its high melting point, low coefficient of thermal expansion, excellent thermal conductivity and electrical conductivity, as well as outstanding antioxidant properties, MoSi_2_ has attracted significant research. It is considered a material of selection for use in a variety of applications, such as high-temperature antioxidant coatings, resistance heating elements, and other functional materials [1,2,3]. Despite these advantages, MoSi_2_ encounters challenges in practical applications. These issues include brittleness at low temperatures, the occurrence of the “PEST” phenomenon in the medium temperature range, and poor creep resistance at high temperatures, all of which hinder its widespread use as a structural material [4,5,6].

In light of the ongoing advancement of modern industry, there is a growing imperative to enhance the mechanical properties and oxidation resistance of MoSi_2_ in order to meet the demands for high-temperature structural materials. The current research focuses on the improvement of these properties through the introduction of a second phase or alloying of the MoSi_2_ matrix with other elements. The primary reinforced phases include SiC, Al_2_O_3_, Si_3_N_4_, WSi_2_ and MoB etc. [7,8,9]. This approach aims to enhance the toughness and creep resistance of MoSi_2_ to a certain extent. For example, nanocrystalline MoSi_2_-SiC composites were fabricated by Patel et al. through liquid silicon infiltration, achieving a hardness of 12 GPa and a fracture toughness of 4.3 MPa·m^1/2^ [10]. Chen et al. produced MoSi_2_-WSi_2_ composites through mechanical activation of Mo, Si, and W powders, followed by SPS. The addition of 20 vol. % WSi_2_ led to a considerable increase in Vickers hardness, reaching a value of 16.47 GPa [11]. Wang et al. synthesized MoSi_2_-Al_2_O_3_ composites by self-propagating combustion, followed by vacuum hot-pressing sintering, achieving high-density structures with well-developed microstructures and outstanding mechanical properties: Vickers hardness of 11.14 GPa, flexural strength of 435 MPa, and fracture toughness of 4.53 MPa·m^1/2^ [12]. In addition to improving the mechanical properties, the oxidation resistance of the material must also be improved. The incorporation of elements with a higher oxygen affinity than Si (i.e., Al, Ti, Zr, and Y) reduced the oxygen flux at the interface between the matrix and the oxide layer, accordingly mitigating the “PEST” phenomenon of MoSi_2_ [13]. To date, a number of studies have been done about the properties of (Mo,Cr)Si_2_ composites [14,15,16,17]. Pan et al. identified chromium (Cr) as a beneficial element for enhancing the oxidation resistance of MoSi_2_ materials by improving the localized hybridization between Si and O [18]. It was observed that Mo-Si-Cr alloys was capable of forming a composite glass phase comprising SiO_2_ and Cr_2_O_3_ during oxidation, which effectively inhibited oxygen diffusion, thus providing effective protection against internal matrix oxidation [19].

MoSi_2_ and its composites are mainly manufactured through the powder metallurgy process. Recent years have witnessed extensive utilization of advanced powder metallurgy techniques, such as hot pressing (HP) [20], mechanical alloying (MA) [21], self-propagating high-temperature synthesis (SHS) [22], and SPS [23], for the synthesis of MoSi_2_ composites. SPS has gained significant attention owing to its fast heating rate, short reaction time, and capacity to achieve high densities. The fundamental principle is based on the exploitation of the thermoelectric effect. The application of high-frequency voltage between the powder sample and the hot electrode induces an electric current within the sample, which is then rapidly heated. The simultaneous application of pressure on the powder enhances particle diffusion and bonding, which in turn facilitates densification [24]. Many research studies have been devoted to the preparation of MoSi_2_-based composites using the SPS technique. Cabouro et al. utilized mechanically activated Mo and Si powders to synthesize dense MoSi_2_ with nanostructures via the SPS technique, and explored the effect of sintering process parameters [25]. Ding et al. prepared MoSi_2_ doped with different B contents using SPS and analyzed the oxidation behavior of the alloyed materials at 1100 ℃ [26]. Huang et al. fabricated a MoSi_2_-3.5 vol. %Si_3_N_4_ composite coating on molybdenum substrate surface using MoSi_2_ and Si_3_N_4_ powders and found a Mo_5_Si_3_ diffusion layer between the coating and the substrate [27].

CrSi_2_, with excellent oxidation resistance, outstanding creep resistance and electronic properties, is commonly used in thermoelectric materials [28,29,30]. Meanwhile, as a transition metal in the same group as Mo, Cr can occupy the Mo sites in the MoSi_2_ lattice [31,32]. Therefore, the goal of this study is to synthesize (Mo,Cr)Si_2_ composites by SPS technology and to investigate the effect of Cr content on the properties of the composites. A comprehensive investigation was conducted into the microstructure, phase composition and mechanical performances of the (Mo,Cr)Si_2_ composites, along with an analysis of their oxidation behavior at 1500 °C.

## 2. Experimental Procedures

### 2.1. Sample Fabrication

**Figure 1:**
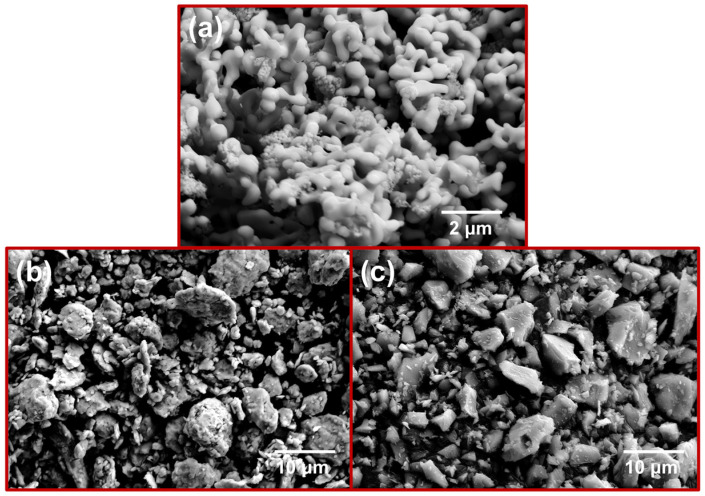
Morphologies of (**a**) as-prepared Mo powders; (**b**) Si powders; (**c**) Cr powders.

The experimental raw materials included ammonium molybdate ((NH4)_2_Mo_4_O_13_, Jinduicheng Molybdenum Co., Ltd., Xi’an, China), carbon black (BR193, Tianjin Ebory Chemical Co., Ltd., Tianjin, China), silicon (Si, 99.99% purity, particle size 5 μm, Beijing Xingrongyuan Technology Co., Ltd., Bejing, China) and chromium (Cr, 99.95% purity, particle size 8 μm, Beijing Xingrongyuan Technology Co., Ltd., Beijing, China) powders. The initial preparation of Mo powder was prepared based on our previously developed method [33]. Briefly, molybdenum trioxide (MoO_3_) was first obtained by calcining ammonium molybdate at 500 °C for 4 h. Subsequently, Mo powder was produced through a two-stage reduction method after ball milling mixing of MoO_3_ with carbon black. Field emission scanning electron microscope (FE-SEM) images in Figure 1 depict the prepared Mo powder alongside the raw materials of Si and Cr powders. In Figure 1a, the Mo powder particles are observed to be round and well-dispersed, with an average particle size of approximately 200–500 nm. The Si powder particles with rounded edges and uneven size distribution are depicted in Figure 1b. Figure 1c exhibits the Cr powder in an irregular particle morphology, with some small particles adhering to larger ones. The particle sizes of the raw materials of Si powder and Cr powder are approximately several micrometers.

**Figure 2:**
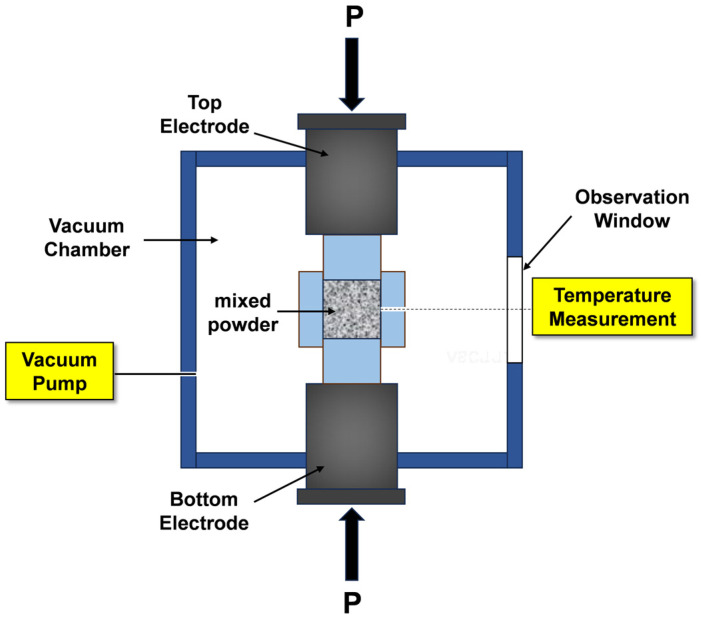
Schematic diagram of spark plasma sintering equipment.

The powder mixtures, formulated with four distinct molar ratios and assigned specific sample names as outlined in Table 1, were prepared using Mo powder, Si powder, and Cr powder. The powders were precisely weighed and subsequently mixed in a high-energy ball mill for 4 h. Anhydrous ethanol was employed as the ball milling medium, with a zirconia ball-to-material ratio of 5:1 and a rotational speed of 200 rpm. The mixed powder was placed in a 20 mm diameter cylindrical graphite mold after drying. Subsequently, the graphite mold containing the sample was inserted into the SPS chamber, and the sintering process was executed under a vacuum atmosphere and high-voltage pulsed direct current at a temperature of 1400 °C. The sintering parameters were 50 MPa pressure, a dwell time of 5 min, and a heating rate of 100 °C/min. The schematic diagram of the SPS device (FIH-452NP, Fuji Electronic Industrial Co., Ltd., Tokyo, Japan) is shown in Figure 2.

### 2.2. Sample Characterization

Upon completion of the sintering process, the resultant samples took the form of discs, each measuring 20 mm in diameter and 4 mm in thickness. The removal of graphite foils from the sample surfaces was conducted through a process of sanding with SiC sandpaper up to 2000 grit, followed by polishing with diamond suspensions to achieve a surface finish that was as smooth as possible. The theoretical densities of (Mo,Cr)Si_2_ composites were derived from the densities of MoSi_2_ (6.28 g/cm^3^) [34] and CrSi_2_ (5.05 g/cm^3^) [35]. The actual densities were obtained through Archimedes’ method.

The mechanical properties of the sintered samples were analyzed by cutting each group into rectangles with dimensions of 12 mm × 4 mm × 3 mm using a diamond wire cutter. Three-point bending experiments were performed on a universal testing machine (WDW-30, Laizhou Lelotte Test Instrument Co., Ltd., Yantai, China) with a loading span of 9 mm and a crosshead speed of 0.5 mm/min. Each composition underwent three measurements to ascertain the average value. Vickers hardness was determined by applying a 1 kg load for 10 s to the surface of the sample using a digital Vickers hardness tester (HVS-30Z, Laizhou Lelotte Test Instrument Co., Ltd. China), with the value calculated by the formula: $Hv=1.854\cdot \frac{f}{d^{2}}$, where *f* denotes the applied load in kg and *d* represents the average of the two diagonal lines of the indentation in mm. Ten randomly selected positions were assessed for Vickers hardness, with the results averaged. The fracture toughness (K_IC_) was computed using the formula proposed by Anstis et al., which measures the crack length under a 5 kg load [36]. The electrical resistivity of the composites was measured using the four-probe method, employing a multifunctional digital four-probe tester, with the results expressed as the mean of three measurements taken for each sample.

The phase compositions of the composites were determined through analysis using an X-ray diffractometer (XRD, TTR III, Rigaku Corporation, Tokyo, Japan) with Cu Kα radiation in the 2θ range of 10–90° at a scanning rate of 0.2 s/step and a step size of 0.02°/step. The microstructures and chemical compositions of the raw material powders, the surfaces of the sintered specimens, the cross-sections, and the oxide layers were characterized by field emission scanning electron microscopy (FE-SEM, ZEISS SUPRA 55, Oberkochen, Germany) with an energy dispersive X-ray spectrometer (EDS).

### 2.3. Oxidation Tests

The high-temperature oxidation experiments were conducted under isothermal conditions at 1500 °C in an air atmosphere. Four samples of each composition in an alumina crucible were placed in a muffle furnace and subsequently oxidized for durations of 2 h, 4 h, 8 h, and 16 h at 1500 °C. Following oxidation, the samples underwent sanding and polishing, and the morphology and thickness of the oxide layer were observed.

## 3. Results and Discussion

### 3.1. Thermodynamic Analysis

During the sintering process, the following primary reactions are hypothesized to occur, based on the composition of the mixed powders, as represented by the following Equations (1)–(3):

(1) $$\begin{array}{c} {\mathrm{Mo}}_{\left(s\right)}+2Si_{\left(s\right)}=\mathrm{MoS}i_{2\left(s\right)} \end{array}$$

(2) $$\begin{array}{c} 5{\mathrm{Mo}}_{\left(s\right)}+3Si_{\left(s\right)}={\mathrm{Mo}}_{5}Si_{3\left(s\right)} \end{array}$$

(3) $$\begin{array}{c} {\mathrm{Cr}}_{\left(s\right)}+2Si_{\left(s\right)}=\mathrm{CrS}i_{2\left(s\right)} \end{array}$$

**Figure 3:**
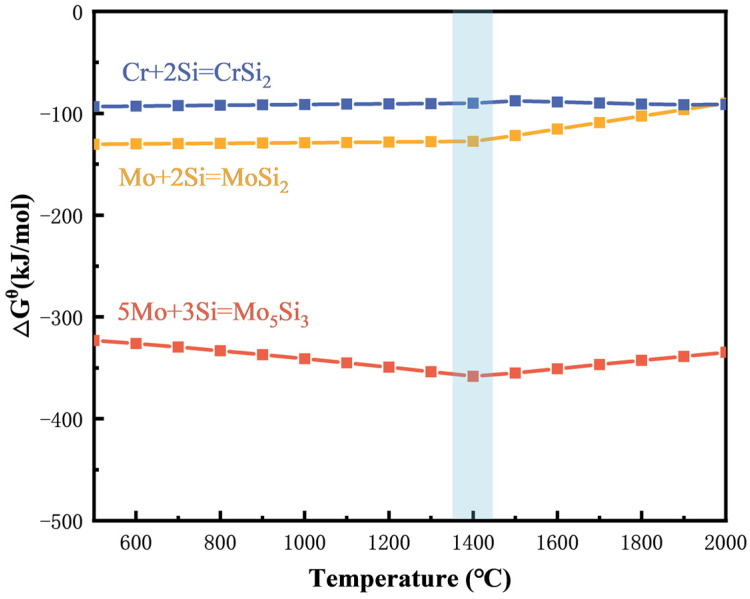
Temperature dependences of the changes of standard Gibbs free energy for Equations (1)–(3).

Thermodynamic analyses of these reactions using HSC Chemistry 6.0 thermodynamic simulation software indicate negative Gibbs free energy (∆G^θ^) changes across all equations (as shown in Figure 3), confirming the thermodynamic feasibility of these reactions under the current experimental conditions. Although the reaction temperature of 1400 °C is slightly below the melting point of Si (1410 °C), since the enthalpy of MoSi_2_ formation is negative (∆H_f_ = −131.8 ± 8.4 kJ mol^−1^) [24], solid Si underwent a transition to a molten state during the exothermic reaction process. This promoted the redistribution of powder particles and subsequent densification of the sample during sintering [37].

### 3.2. The XRD Patterns and Microstructures of the Sintered Composites

**Figure 4:**
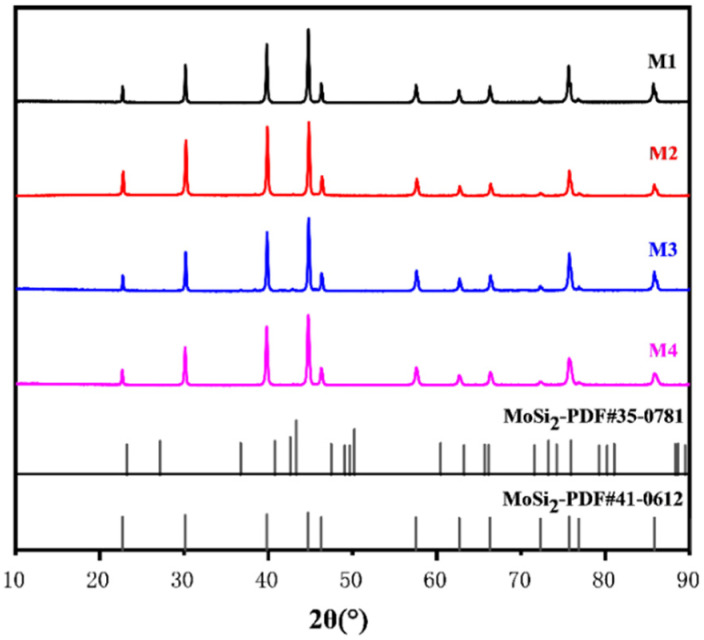
XRD patterns of the as-sintered MoSi_2_ composites with different Cr content.

**Figure 5:**
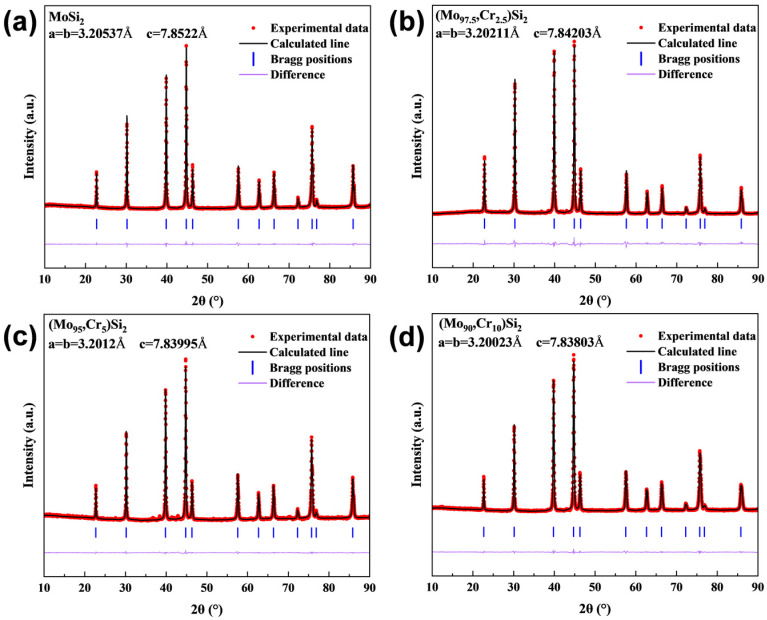
XRD patterns of sintered (**a**) M1, (**b**) M2, (**c**) M3, and (**d**) M4 samples after Rietveld refinement.

The XRD spectra of the samples with varying Cr contents sintered at 1400 °C are depicted in Figure 4, and all results showed diffraction peaks of a single phase. The formation of MoSi_2_ from the mixed powders was observed during the sintering process, with the complete consumption of the raw materials Mo, Si, and Cr. According to previous studies, the preparation of MoSi_2_-based composites often leads to the formation of Mo_5_Si_3_ due to MoSi_2_ oxidation during the reaction process [38,39]. However, Mo_5_Si_3_ was not found in the XRD results of the sintered samples in this study. It is pertinent to note that the characteristic peaks of the CrSi_2_ phase were absent in the XRD patterns of Cr-doped samples. The XRD data were subjected to Rietveld refinement using HighScore (version 5.2) software, and Figure 5 displays the lattice parameters of each sintered sample. The lattice parameters decrease in a progressive manner with Cr doping in the MoSi_2_ matrix. Given that Cr has a smaller atomic radius than Mo, it is plausible that solid solution substitution occurred during the reaction sintering process, whereby Cr atoms replaced some Mo atoms in the MoSi_2_ lattice, thereby reducing its lattice parameters.

**Figure 6:**
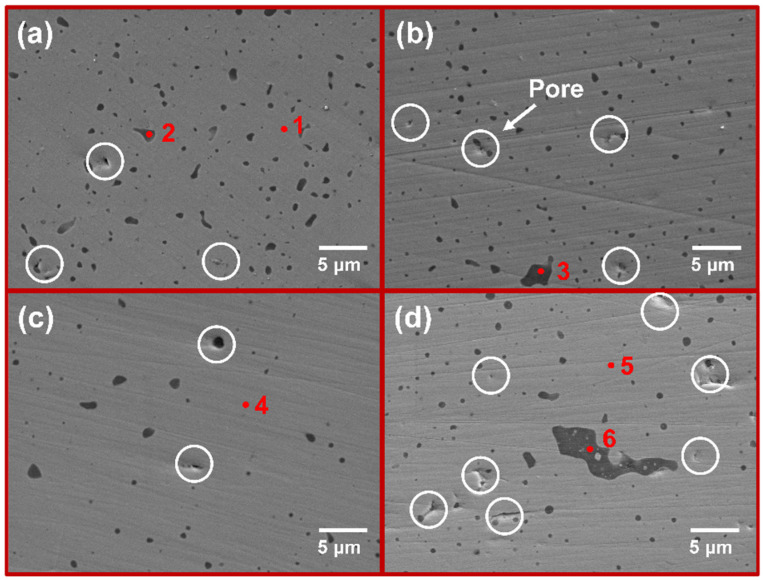
The morphology images of sintered products: (**a**) M1; (**b**) M2; (**c**) M3; (**d**) M4.

Figure 6 shows the surface morphology of each sintered sample, while Table 2 presents the elemental compositions. All samples have a relatively uniform and dense surface with only a few small pores. As illustrated in Figure 6a, a uniformly distributed dark second phase is observed within the MoSi_2_ matrix. The chemical analysis via EDS reveals that the phase where point 1 is located is mainly composed of Si and O. Based on the atomic ratios observed, it can be inferred that this second phase should be SiO_2_. This is due to the unavoidable adsorption of some oxygen on the powder surface during the preparation process. Therefore, during reaction sintering, Si combines with the residual O to form SiO_2_. Despite this, no diffraction peaks corresponding to SiO_2_ were observed, which may be due to its amorphous state and content of less than 5 wt.%. [40]. As the Cr content is increased, the surface morphology remains almost the same. As illustrated in Figure 6d, the surface structure of the M4 sample with 10 mol. % CrSi_2_ addition is significantly deteriorated. The pore shapes observed in the M4 sample are more irregular in comparison to the circular pores present in the other samples. As stated by Frankwick et al., the solubility of Cr in MoSi_2_ after annealing at 1300 °C is approximately 3 at. % [14]. However, the addition of Cr (3.3 at. %) in the M4 sample slightly exceeds this range. Consequently, it can be concluded that Cr was unable to fully solid-solve into the MoSi_2_ matrix. Meanwhile, due to the strong oxygen affinity of Cr, some of it is oxidized to Cr_2_O_3_, which together with SiO_2_ forms the second phase, as evidenced by point 3 in Table 2. However, due to the low content of Cr in the second phase, it cannot be reflected in XRD.

### 3.3. Sintering Densification

**Figure 7:**
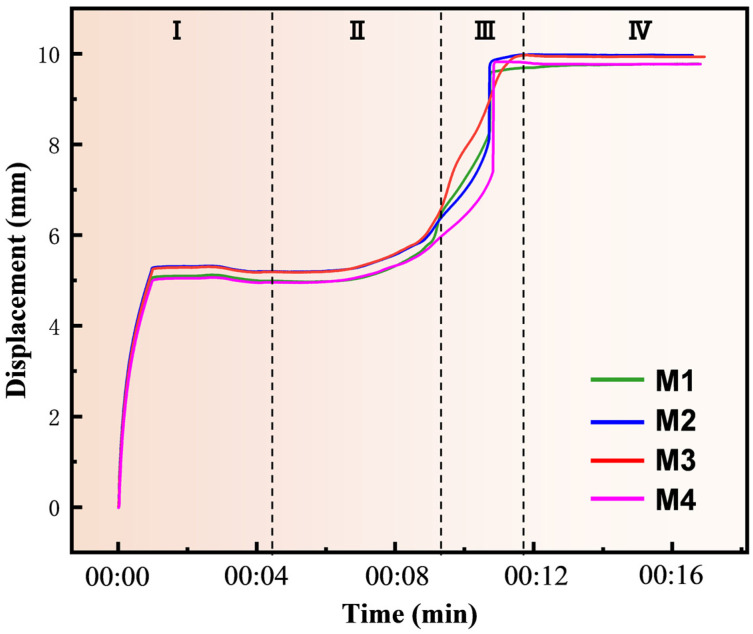
The piston displacement versus time curves of samples with different Cr contents sintered at 1400 °C with a 5 min dwell time.

Figure 7 shows the variation of the shrinkage displacement with time during the sintering process for four groups of samples. The alteration of displacement in the samples serves as a significant indicator of reaction sintering, with changes in displacement direction indicating specimen contraction or expansion. The reaction process can be separated into discrete stages. At the initial stage of the heating phase, the displacement curve shifts slightly in the negative direction due to the thermal expansion of powder particles. Subsequently, in the second stage, the specimen undergoes gradual shrinkage as the temperature rises, which is due to the compaction of the powder mixture. In the third stage, at the temperature conducive to in situ reaction, accelerated Si diffusion promotes MoSi_2_ formation, leading to rapid densification and visible specimen contraction. This contraction is evident from a sharp increase in displacement, reflecting sample volume changes both before and after the reaction. Finally, in the fourth stage, after the reaction is complete, the displacement profile is almost constant. The primary distinction among the four samples lies in the varied rates of contraction during the third stage, potentially influenced by differences in reaction rate stemming from varying component compositions.

**Figure 8:**
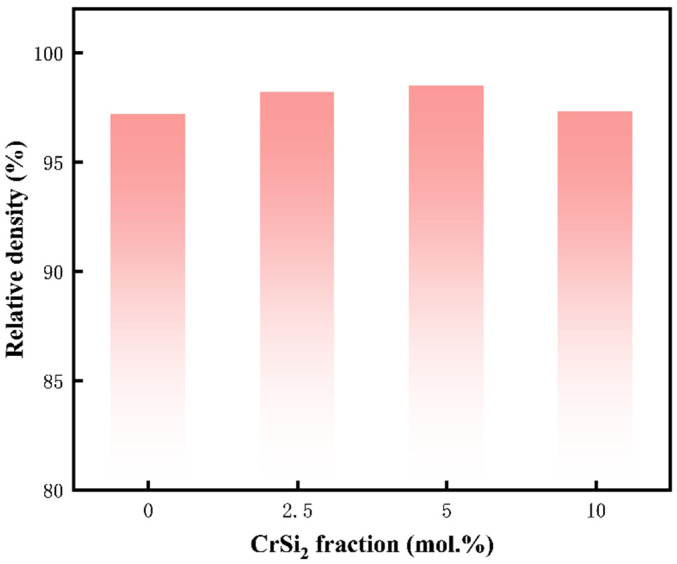
Relative density–Cr content histogram of the as-sintered composites.

It is well recognized that fine powders exhibit higher sintering activity owing to the elevated surface energy, which promotes consolidation and grain growth [41]. The self-produced Mo powder utilized in this study features smaller particle sizes, therefore contributing to the densification of the (Mo,Cr)Si_2_ composites. Figure 8 presents the relative densities of four (Mo,Cr)Si_2_ composites prepared with different Cr additions. The sintered samples of each component have a higher density. It is apparent that the relative density of the M3 sample reached 98.5% with the incorporation of Cr into the MoSi_2_ matrix. Nevertheless, upon reaching a CrSi_2_ content of 10 mol. %, there is a slight decrease in the relative density of the composite, as corroborated by SEM images.

### 3.4. Properties of the Sintered Composites

**Figure 9:**
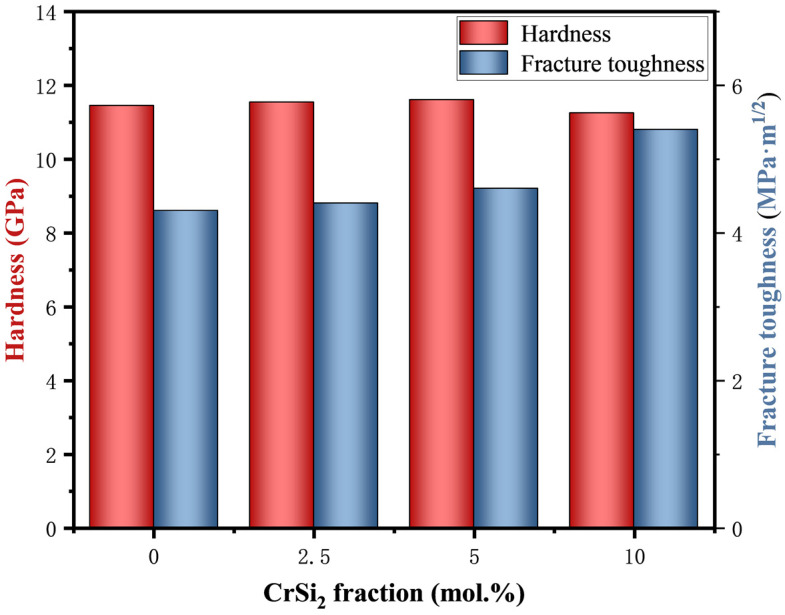
Hardness (Hv) and indentation fracture toughness (K_IC_) of as-sintered composites versus Cr content.

The Vickers hardness and fracture toughness of (Mo,Cr)Si_2_ sintered samples are illustrated in Figure 9. As seen in the figure, the mechanical properties of (Mo,Cr)Si_2_ composites are markedly superior to those of pure MoSi_2_ materials. In the study by Harada et al. where Cr was introduced into MoSi_2_, increasing the Cr addition up to the limit of solubility resulted in a slight decrease in microhardness [42]. Through solid solution strengthening, the hardness of the composite material increases as the Cr content is added, but the overall change is not significant. The highest Vickers hardness of 11.6 GPa was exhibited by the M3 sample. However, when the CrSi_2_ content was increased to 10 mol. %, a phenomenon of inhomogeneous distribution of the second phase was observed, accompanied by the appearance of agglomerates and pores. Meanwhile, the hardness of the M4 sample decreases as its densification degree decreases. Moreover, the fracture toughness of the sintered composites enhanced gradually with the addition of Cr, particularly with a notable sudden increase observed in the fracture toughness of M4 sample.

**Figure 10:**
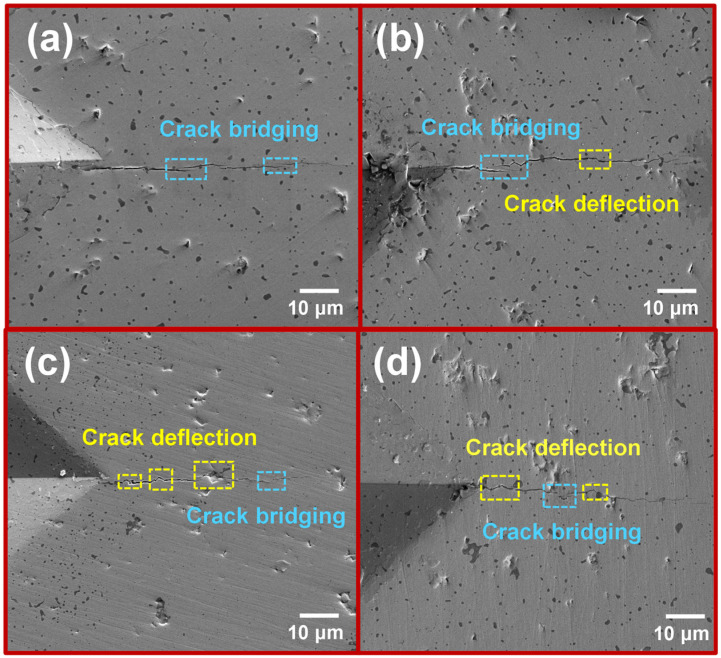
Crack propagation path of MoSi_2_ composites with different Cr contents: (**a**) M1; (**b**) M2; (**c**) M3; (**d**) M4.

To gain further insight into the variation in fracture toughness of (Mo,Cr)Si_2_ composites, the paths of crack extension were subjected to detailed investigation. The cracks produced by Vickers indentation for each set of specimens are depicted in Figure 10. It can be seen in Figure 10a that the cracks of the M1 sample extend along a straight-line direction, indicating the presence of detectable crack bridging, albeit predominantly in a straight direction. In Figure 10b, the sample containing Cr also experiences crack deflection during extension, without significant hindrance to crack propagation. As indicated in Figure 10c, a further-increasing Cr content leads to a more complex extension path, which promotes the branching of cracks. Furthermore, Figure 10d suggests that the M4 sample further enhances its fracture toughness due to the presence of the composite second phase of Cr_2_O_3_ and SiO_2_. Both crack bridging and deflection serve to decrease the driving forces for crack propagation, accordingly increasing the energy required for crack propagation and achieving toughening effects [43].

**Figure 11:**
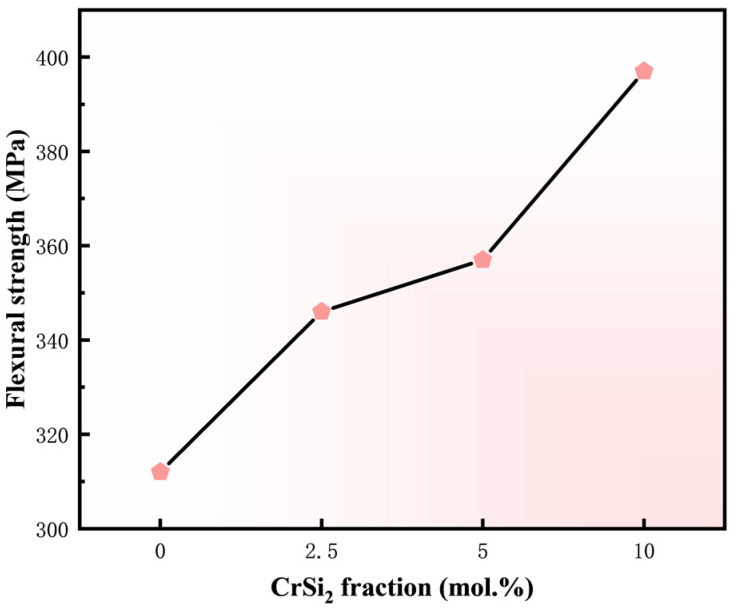
Flexural strength of as-sintered composites versus Cr content.

**Figure 12:**
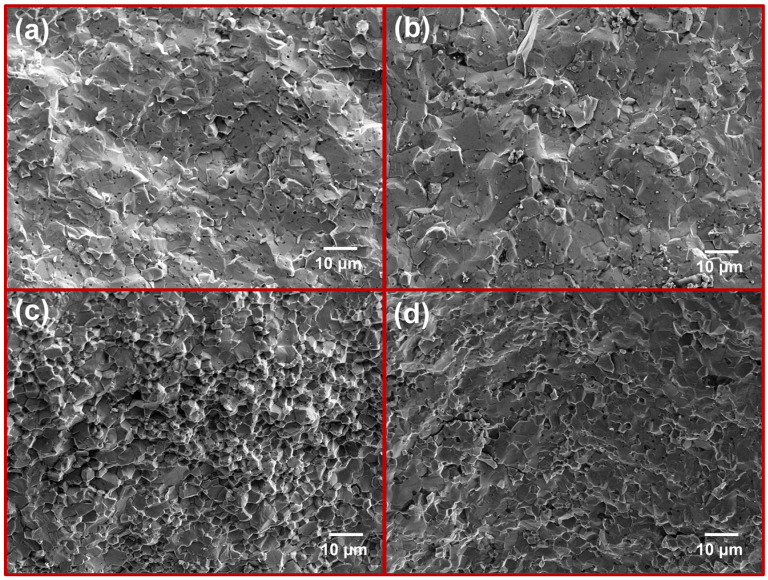
Fracture morphologies of MoSi_2_ composites with various Cr contents: (**a**) M1; (**b**) M2; (**c**) M3; (**d**) M4.

**Figure 13:**
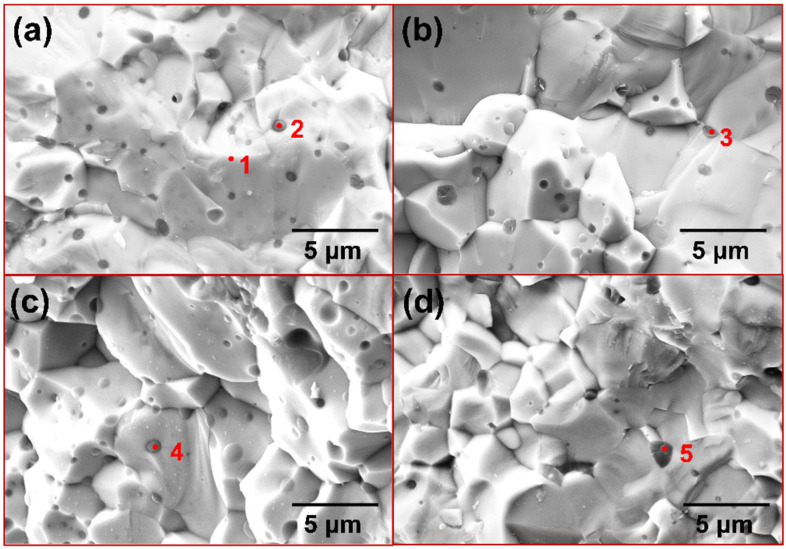
Fracture morphologies with higher magnification: (**a**) M1; (**b**) M2; (**c**) M3; (**d**) M4.

The variation of flexural strength of the sintered samples with Cr content is presented in Figure 11. The M4 sample achieves the highest flexural strength of 397 MPa. Figure 12 presents the microstructures of the fracture surfaces of four sintered samples, which are typically brittle due to the inherent properties of MoSi_2_. In Figure 12a, the fracture mode of the pure MoSi_2_ sample displays a typical transgranular pattern. The fracture surface morphology appears relatively flat, with distinct cleavage steps and no evident signs of ductility or deformation. In addition, there are numerous minute pores in the matrix that are the cause of the reduction in fracture toughness of the specimen. In Figure 12b, the incorporation of a modest quantity of the Cr element markedly reduces the number of pores, while the predominant fracture mode remains transgranular. As the Cr content increases, Figure 12c illustrates a gradual transition towards intergranular fracture. In Figure 12d, the M4 sample features a second phase at the grain boundaries, resulting in a mixed intergranular and transgranular fracture mode. Figure 13 is the SEM image of the fracture surface with a higher magnification. It can be clearly seen that the dark phase is distributed throughout the matrix. According to the EDS results in Table 3, it can be concluded that the dark phase is the second phase containing oxygen element. As is well known, grain size has a considerable impact on the mechanical performances of composites, especially strength [44]. From the cross section of the sample, it is possible to see that the grain size of the composite material is markedly smaller than that of the pure MoSi_2_. It is thus concluded that the introduction of Cr markedly improves the mechanical properties of MoSi_2_, with particular emphasis on the superior overall mechanical characteristics observed in the M3 sample.

Electrical resistivity is one of the important properties of composite materials for resistance heating elements. Table 4 lists the electrical resistivity of the samples with different compositions. As the CrSi_2_ content increases from 0 mol. % to 10 mol. %, the resistivity of the (Mo,Cr)Si_2_ composites increases from 16.9 to 24.7 µΩ·cm. It can be seen that the resistivity values of the sintered specimens show a consistent increase in conjunction with elevated CrSi_2_ content. According to Nishida et al., the resistivity of pure CrSi_2_ is 190 µΩ·cm [45], which is much higher than that of the M1 sample MoSi_2_ and leads to the increase in electrical resistivity.

### 3.5. Oxidation Behavior at 1500 °C

The oxidation behavior of four samples with variable Cr contents was investigated at 1500 °C for different durations. The potential reactions of (Mo,Cr)Si_2_ composites during oxidation are outlined as follows [19,46]:

(4) $$\begin{array}{c} 2/7\mathrm{MoS}i_{2\left(s\right)}+O_{2\left(g\right)}=2/7\mathrm{Mo}O_{3\left(s\right)}+4/7\mathrm{Si}O_{2(s)} \end{array}$$

(5) $$\begin{array}{c} 5/7\mathrm{MoS}i_{2\left(s\right)}+O_{2\left(g\right)}={1/7\mathrm{Mo}}_{5}Si_{3\left(s\right)}+\mathrm{Si}O_{2(s)} \end{array}$$

(6) $$\begin{array}{c} \frac{4}{11\mathrm{CrS}i_{2\left(s\right)}}+O_{2\left(g\right)}=\frac{2}{11{\mathrm{Cr}}_{2}O_{3\left(s\right)}}+\frac{8}{11\mathrm{Si}O_{2\left(s\right)}} \end{array}$$

**Figure 14:**
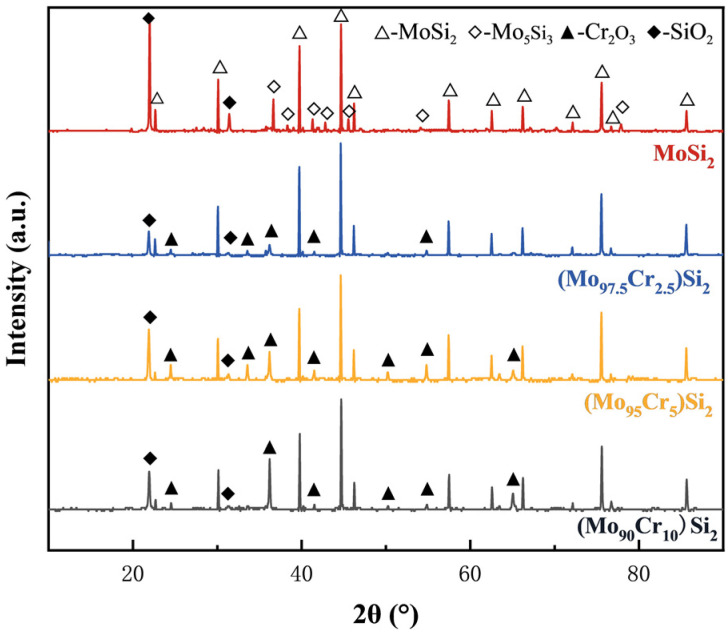
XRD patterns of MoSi_2_ composites with different Cr contents after oxidation at 1500 °C for 16 h.

The XRD results of the surface oxide layers of the four samples after oxidizing at 1500 °C for 16 h are displayed in Figure 14. The results demonstrate the presence of the SiO_2_ phase on the surface of all samples. Additionally, the presence of Cr_2_O_3_ was confirmed in samples containing elemental Cr. It is noteworthy that diffraction peaks of the MoSi_2_ phase are discernible in all of the oxidized specimens. This phenomenon is likely attributable to the thinness of the oxide layer, which permits X-ray diffraction analysis to detect the phase composition up to ten microns beneath the surface, thus revealing the matrix phase characteristic peaks [47].

Based on the XRD results, the oxidation mechanism at 1500 °C can be inferred as follows: the oxidation process first initiates reaction (4) to produce MoO_3_ and SiO_2_. However, the high temperature volatilization of MoO_3_ causes the surface to form pores. Simultaneously, the SiO_2_ layer is too thin to completely cover the surface of the specimen, thereby failing to isolate the oxygen, which results in the further oxidation of the (Mo,Cr)Si_2_. As the oxidation continues, the surface of the substrate forms a high-density SiO_2_ protective film. Because of the low oxygen diffusion coefficient of SiO_2_, it effectively impedes oxygen ingress, therefore safeguarding the substrate from continued oxidation. Furthermore, the viscous flow of SiO_2_ can repair surface defects, such as pores caused by MoO_3_ volatilization [48]. As the partial pressure of oxygen at the SiO_2_-MoSi_2_ interface decreases, it becomes more likely that reaction (5) will take place. As a result, a minor quantity of the Mo_5_Si_3_ phase was detected in the M1 sample. In contrast, the Mo_5_Si_3_ phase disappeared upon the introduction of the Cr element, with the simultaneous generation of Cr_2_O_3_. This introduction of Cr leads to the formation of Cr_2_O_3_, which consumes oxygen and reduces MoSi_2_ depletion within the matrix, thereby preventing the formation of Mo_5_Si_3_. In reaction (6), two protective oxide layers, Cr_2_O_3_ and SiO_2_, are simultaneously generated. The aforementioned oxide layers have high resistance to volatilization and oxygen diffusion, which promotes the stability of the oxide scale at higher temperatures. [49].

**Figure 15:**
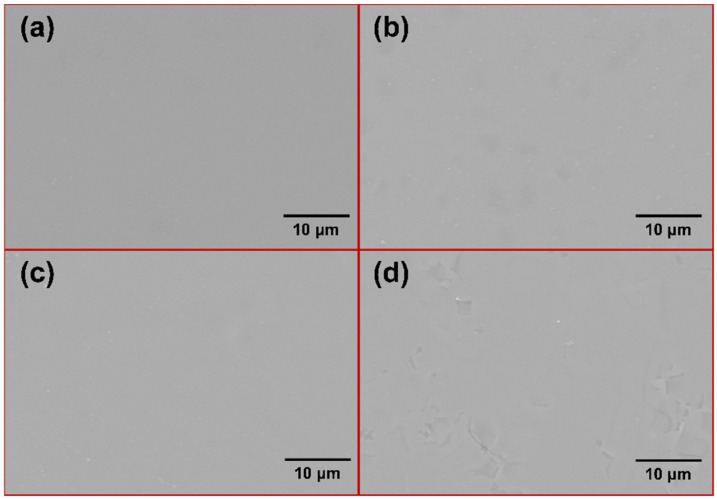
The surface morphology of four oxidized samples: (**a**) M1; (**b**) M2; (**c**) M3; (**d**) M4.

**Figure 16:**
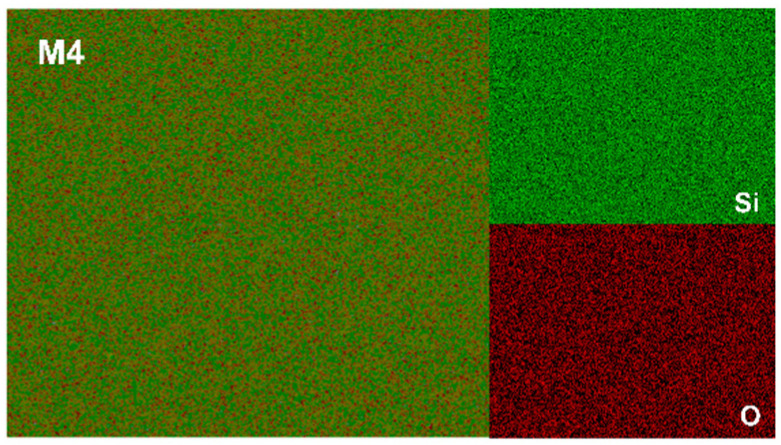
The mapping analysis of the oxide surface of M4 samples.

Figure 15 shows the surface morphologies of the four samples after 16 h of oxidation, and it can be seen that the surfaces of the samples are covered with the uniform and flat glass phase. The mapping analyses of the four sets of samples are consistent and indicate that they all consist of two elements, Si and O, with uniform distributions, as shown in Figure 16. It is worth noting that Figure 15d is slightly different, in that the presence of other shapes can be faintly seen, which may be due to the high proportion of Cr_2_O_3_ observed through the thin SiO_2_ layer.

**Figure 17:**
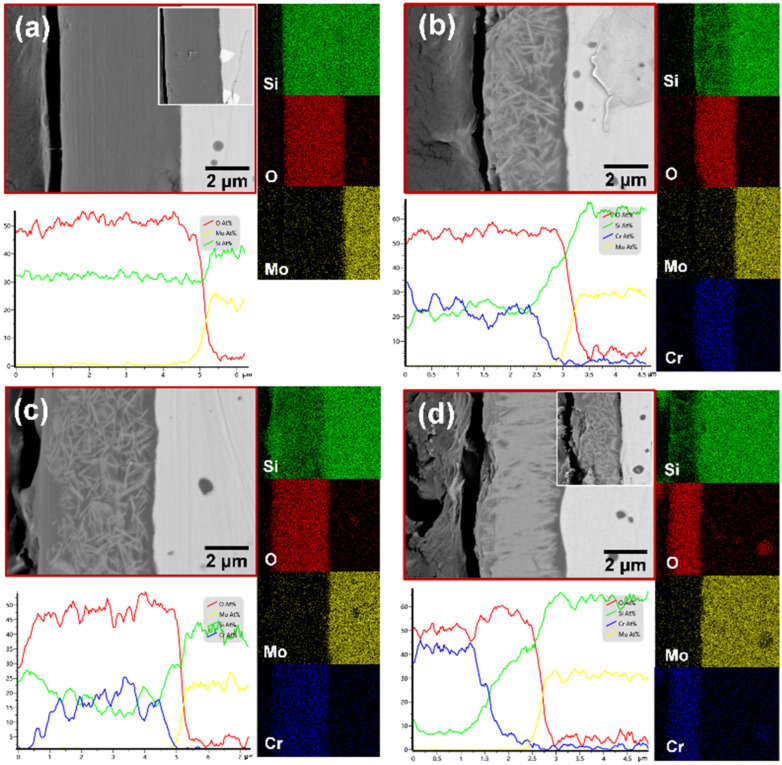
Cross-section images, EDS line scanning, and mapping analyses of four oxidized samples: (**a**) M1; (**b**) M2; (**c**) M3; (**d**) M4.

In Figure 17, the cross sections of four samples after 16 h of oxidation are depicted. Based on the oxide layer structure and elemental distribution diagrams, it is evident that the surfaces of all oxidized samples exhibit a uniform and dense glassy oxide layer. In addition, the analysis of elemental distributions across the samples indicates the presences of Si, O, and Cr within the oxide layer. The minimal presence of oxygen distribution within the matrix provides compelling evidence that the oxide layer acts as an effective barrier against inward oxygen diffusion, thus shielding the matrix from further oxidation. Importantly, the absence of the Mo element in the oxide layer indicates the complete volatilization of the oxidation product MoO_3_ at high temperatures.

In Figure 17a, it can be found that the surface of the oxide layer of the M1 sample exhibits a flat morphology, with a well-bonded interface between the oxide layer and the substrate. Additionally, a white phase is discernible at the interface between the matrix and the oxide scale. As illustrated in the inset of Figure 17a, this phase has been identified as Mo_5_Si_3_ through EDS elemental analysis. Figure 17b depicts the M2 sample, which has been doped with Cr element and similarly presents a flat surface devoid of visible crevices. It is notable that the oxide scale is composed of two parts: a flat and dense layer of SiO_2_ in the outer side, and strips of Cr_2_O_3_ surrounded by SiO_2_ in the inner side. Figure 17c demonstrates that, as the Cr content is added, the thickness of the oxide layer also rises, along with the proportion of Cr_2_O_3_. Upon oxidation of the M4 sample in Figure 17d, the oxide layer exhibits an uneven thickness, a non-flat surface, and the emergence of cracks at the substrate interface. Throughout the oxidation process, oxygen can quickly penetrate into the interior of the sample via cracks, which impairs the protective function of the SiO_2_ oxide layer. This phenomenon is influenced by the substantial disparity in thermal expansion coefficients between Cr_2_O_3_ (9.6 × 10^−6^/K) and MoSi_2_ (8.1 × 10^−6^/K) [50,51]. During the cooling process, the thermal stresses can induce cracking at the interface between the oxide layer and the substrate, as illustrated in the inset of Figure 17d. This issue may arise from the presence of Cr_2_O_3_ in the oxide layer, which inhibits complete encapsulation by SiO_2_ and weakens the interfacial bonding, consequently causing detachment of the oxide layer from the substrate. As a result, these structural imperfections, such as cracks, act as pathways for oxygen diffusion, leading to internal oxidation.

**Figure 18:**
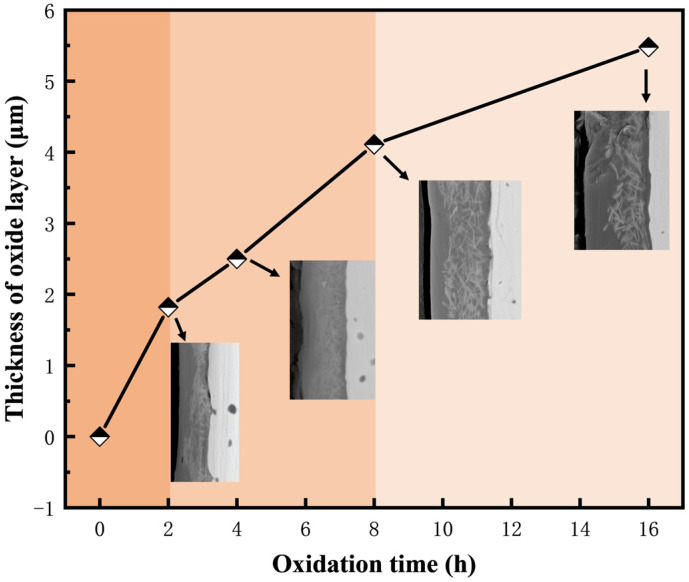
The average thickness of the oxide layer of the M3 samples after an isothermal oxidation test for different times.

**Figure 19:**
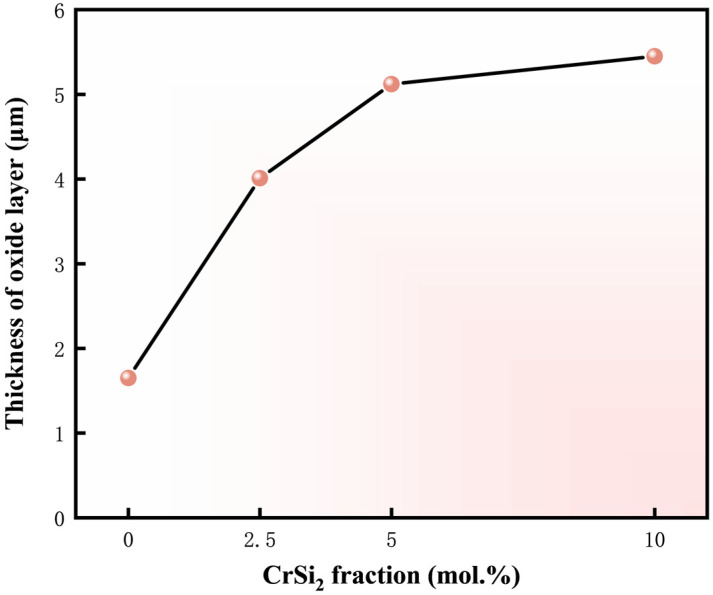
The average thickness of the oxide layer of four samples after an isothermal oxidation test for 16 h.

Figure 18 depicts the variation of the oxide film thickness of the M3 sample with oxidation time. It is noticeable that the oxide film becomes thicker as the oxidation time increases. During the initial oxidation stage, rapid oxidation of the sample surface causes a significant increase in the oxide film thickness. At this stage, the oxidation products are insufficient to fully encapsulate the substrate surface, allowing the oxidation reaction to continue and form a protective glass layer. As the oxidation time proceeds, the slope of the thickness curve gradually decreases by the gradual establishment of a complete oxide scale, which prevents the inward diffusion of oxygen. Meanwhile, Figure 19 shows that following the identical oxidation duration, the oxide layer of the sample with Cr addition exhibits a marked thickening. This is attributed to the oxidation of CrSi_2_ yielding SiO_2_ and Cr_2_O_3_ oxides, with increasing Cr content resulting in greater Cr_2_O_3_ production and consequent oxide layer thickening. But, as the Cr content is further enhanced, the growth rate of the oxide film thickness gradually slows down. The results of many studies have shown that the composite glass layer of SiO_2_ and Cr_2_O_3_ has good stability and low oxygen diffusion [52,53]. In summary, (Mo,Cr)Si_2_ composites can form a protective SiO_2_-Cr_2_O_3_ oxide layer, which provides better protection to the matrix at high temperatures.

## 4. Conclusions

This investigation was focused on the synthesis of (Mo,Cr)Si_2_ composites with varying Cr compositions through SPS at 1400 °C, with the raw materials of Mo powder, Si powder, and Cr powder. This study presents systematic investigations on the microstructural features, phase compositions, mechanical properties and high-temperature oxidation resistances of samples with CrSi_2_ contents of 0, 2.5, 5, and 10 mol. %. The following conclusions can be drawn from this investigation:

(1) In this study, (Mo,Cr)Si_2_ composites were prepared at 1400 °C using SPS technology. A moderate amount of Cr can be solidly dissolved into the MoSi_2_ matrix. Simultaneously, a small amount of oxide is distributed as a second phase in the matrix.
(2) The addition of the Cr element has the potential to enhance the mechanical performance of the composites. The (Mo_95_,Cr_5_)Si_2_ samples exhibit the most favorable comprehensive mechanical properties, with a Vickers hardness of 11.6 GPa, a fracture toughness of 4.6 MPa·m^1/2^, and a flexural strength of 397 MPa.
(3) During the oxidation at 1500 °C, the oxidation products of Cr-added samples produce Cr_2_O_3_ alongside SiO_2_. Nevertheless, the mismatch in thermal expansion coefficients between Cr_2_O_3_ and the matrix can cause Cr_2_O_3_ to delaminate and separate from the oxide layer, resulting in layer failure.

## Figures and Tables

**Table 1:** Compositions and designations of different composites.

Sample Number	MoSi_2_/mol. %	CrSi_2_/mol. %
M1	100	0
M2	97.5	2.5
M3	95	5
M4	90	10

**Table 2:** Energy spectrum data of various points in the as-sintered composites.

Point	Element (at. %)			
	Mo	Si	O	Cr
1	32.4	67.6		
2	7.0	39.9	53.2	
3		39.0	61.0	
4	32.1	67.1		0.9
5	29.9	67.7		2.3
6		41.0	58.3	0.6

**Table 3:** Energy spectrum data of various points in the fracture surface.

Point	Element (wt. %)			
	Mo	Si	O	Cr
1	60.5	39.5		
2	33.0	40.1	26.9	
3	39.4	40.5	19.5	0.6
4	23.2	57.3	17.1	2.4
5	28.0	47.0	24.1	0.9

**Table 4:** Electrical resistivity of the as-sintered composites.

Sample Number	Electrical Resistivity (µΩ·cm)
M1	16.9
M2	18.7
M3	21.5
M4	24.7

## Data Availability

The raw data supporting the conclusions of this article will be made available by the authors on request.
